# Effects of Ketone Bodies on Brain Metabolism and Function in Neurodegenerative Diseases

**DOI:** 10.3390/ijms21228767

**Published:** 2020-11-20

**Authors:** Nicole Jacqueline Jensen, Helena Zander Wodschow, Malin Nilsson, Jørgen Rungby

**Affiliations:** 1 Department of Endocrinology, Bispebjerg University Hospital, 2400 Copenhagen, Denmark; helenazw@outlook.com (H.Z.W.); malin.sofia.desiree.nilsson@regionh.dk (M.N.); joergen.rungby@regionh.dk (J.R.); 2 Copenhagen Center for Translational Research, Copenhagen University Hospital, Bispebjerg and Frederiksberg, 2400 Copenhagen, Denmark

**Keywords:** ketone bodies, neurodegeneration, cognition, cerebral metabolism, ketogenic diet, ketone supplements, astrocytes, SGLT-2 inhibitors

## Abstract

Under normal physiological conditions the brain primarily utilizes glucose for ATP generation. However, in situations where glucose is sparse, e.g., during prolonged fasting, ketone bodies become an important energy source for the brain. The brain’s utilization of ketones seems to depend mainly on the concentration in the blood, thus many dietary approaches such as ketogenic diets, ingestion of ketogenic medium-chain fatty acids or exogenous ketones, facilitate significant changes in the brain’s metabolism. Therefore, these approaches may ameliorate the energy crisis in neurodegenerative diseases, which are characterized by a deterioration of the brain’s glucose metabolism, providing a therapeutic advantage in these diseases. Most clinical studies examining the neuroprotective role of ketone bodies have been conducted in patients with Alzheimer’s disease, where brain imaging studies support the notion of enhancing brain energy metabolism with ketones. Likewise, a few studies show modest functional improvements in patients with Parkinson’s disease and cognitive benefits in patients with—or at risk of—Alzheimer’s disease after ketogenic interventions. Here, we summarize current knowledge on how ketogenic interventions support brain metabolism and discuss the therapeutic role of ketones in neurodegenerative disease, emphasizing clinical data.

## 1. Introduction

The human brain requires a significant amount of energy for normal brain function and accounts for about 20% of the body’s total energy expenditure at rest, despite the fact that the brain only represents ~2% of the total body weight [1]. Most of the brain’s energy consumption is derived from glucose oxidation and is predominantly used to support synaptic transmission, including the maintenance of ion gradients [2,3]. In addition to the energy requirements for neuronal signalling, other cellular processes such as remodelling of the cytoskeleton, synthesis of phospholipids, and axonal transport, also require ATP [4]. Therefore, an adequate and continuous supply of energy is necessary to maintain brain cellular function since only a limited amount of glycogen is stored inside the brain [5]. This is emphasized in pathological conditions where brain metabolism is disturbed, e.g., in glucose transporter type 1 (GLUT-1) deficiency resulting in impaired cerebral glucose uptake, where clinical symptoms may manifest as seizures, movement disorders, and cognitive impairments [6].

While the brain primarily relies on glucose as the main fuel, other substrates may contribute to metabolism, especially when glucose supply is restricted or inadequate, e.g., during fasting and low carbohydrate diets [7,8]. Ketone bodies, together with lactate, are the main alternative fuels for the brain and both are able to cross the blood–brain barrier through monocarboxylate transporters (MCTs) in endothelial cells and astroglia [9]. Plasma ketone levels are usually low after an overnight fast (<0.5 mM) and contribute to less than 5% of the brain’s metabolism [10]. However, during prolonged fasting (5–6 weeks), ketone body levels rise significantly and are able to contribute almost 60% of the brain’s energy requirement, thereby replacing glucose as the main fuel [7]. Ketonemia can be achieved in non-fasting states by ketogenic diets or by the ingestion of supplements in the form of ketogenic medium-chain fatty acids (MCFA) or exogenous ketone esters or salts. When plasma levels of ketone bodies are raised either by fasting, diet or infusion, they are transported to the brain and metabolized in a concentration-dependent manner [10], consequently offering a strategy to alter or enhance cerebral metabolism in disorders with a disturbed glucose metabolism.

The ketogenic diet was developed in the 1920s as a treatment for epilepsy due to early observations of an antiseizure effect [11]. During the last decade, the interest in ketogenic diets and other ketogenic treatments has increased rapidly, and ketone bodies are now thought to be a potential therapeutic strategy in many disorders such as cancer, diabetes, cardiovascular disease, and neurodegeneration [12,13]. A growing number of preclinical and clinical studies exist on different ketogenic approaches in neurodegenerative diseases, especially Alzheimer’s disease (AD). One common feature in diseases characterized by neurodegeneration is disruption of the brain’s energy metabolism. In AD, Parkinson’s disease (PD), amyotrophic lateral sclerosis (ALS), and Huntington disease (HD), glucose hypometabolism in affected brain regions is prominent, which correlates to disease severity [14,15,16,17]. Therefore, approaches which support brain energetics, such as ketone treatments, may slow disease progression or even delay or prevent disease onset if initiated early enough. A comprehensive review on the status and prospects of different energetic strategies in neurodegeneration was recently published [18].

In this review, we focus on different ketogenic approaches and describe the metabolic actions of ketone bodies in the brain and highlight key clinical data that support the neuroprotective effects of ketones in diseases of neurodegeneration.

## 2. Ketone Bodies Reaching the Brain

During fasting, free fatty acids are mobilized from adipocytes and transported to the liver where they contribute to the synthesis of ketone bodies [19]. This process is dependent on low insulin levels, which enhances lipolysis in white adipose tissue due to the suppressed insulin-induced inhibition on hormone sensitive lipase. In hepatocytes, they subsequently undergo beta-oxidation that may initiate ketogenesis [19]. Due to the need of transporters for the entry of long chain fatty acids to the mitochondrial matrix, this could be a limiting step for ketosis during a ketogenic diet. Interestingly, MCFA are not dependent on the transporter protein for mitochondrial entry [19].

### 2.1. The Synthesis of Ketone Bodies in the Liver

**Figure 1:**
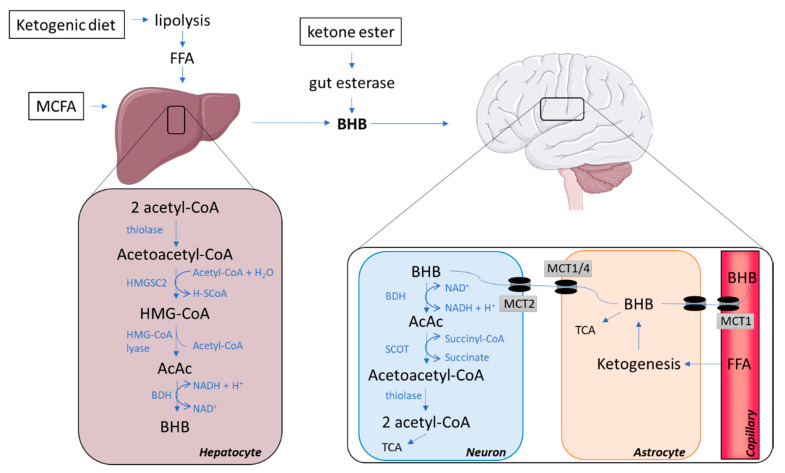
Pathways involved in synthesis and catabolism of ketone bodies. AcAc, acetoacetate; Acetyl-CoA, acetyl coenzyme A; BHB, beta-hydroxybutyrate; BHD, beta-hydroxybutyrate dehydrogenase; FFA, free fatty acids; HMG-CoA, 3-hydroxy-3-methylglutaryl-CoA; HMGCS2, 3-Hydroxy-3-Methylglutaryl-CoA Synthase 2; MCFA, medium-chain fatty acids; MCT, monocarboxylate transporter; SCOT, succinyl-CoA:3-ketoacid Coenzyme A transferase; TCA, tricarboxylic acid cycle.

Hepatocytes are the main site for the synthesis of ketones but the process may also occur, albeit to a minor extent, in the kidneys and in astrocytes [20]. A high level of acetyl coenzyme A (acetyl-CoA) is essential for ketogenesis, and is obtained mainly through a high supply of free fatty acids, which can lead to a surplus production of acetyl-CoA not entering the tricarboxylic (TCA) cycle for ATP generation but leading to the formation of ketone bodies [19]. Hence, ketone bodies are mainly derived from fatty acids via beta-oxidation; however, amino acids (particularly leucine) may contribute about 4% of the total ketone body production in the post-absorptive state [20]. Ketogenesis requires the action of at least three enzymes: (I) mitochondrial Acetoacetyl-CoA thiolase, (II) mitochondrial 3-hydroxy-3-methylglutaryl-CoA (HMG-CoA) synthase (HMGCS2), and (III) HMG-CoA lyase, which form the ketone body, acetoacetate (Figure 1; hepatocyte).

The initial step of ketogenesis is the formation of acetoacetyl-CoA, which is derived from two acetyl-CoA by the enzyme thiolase. The newly produced acetoacetyl-CoA is then combined with another acetyl-CoA via a reaction with HMGCS2, hence forming HMG-CoA. This reaction is the rate-limiting step, which is regulated by insulin and glucagon, which inhibit and facilitate *Hmgcs2* transcription via forkhead transcriptional factor FOXA2 [20]. The enzyme HMG-CoA lyase subsequently removes an acetyl-CoA from HMG-CoA to produce the ketone body acetoacetate, which can either undergo spontaneous degradation to acetone (another ketone body with minimal metabolic contribution) or form the ketone body beta-hydroxybutyrate (BHB) [20], the most abundant ketone body in the human circulation [21]. This occurs through an enzymatic reaction with beta-hydroxybutyrate dehydrogenase (BDH), requiring a hydrogen donated from the NAD+/NADH couple. Acetoacetate and BHB are not utilized in the liver, as it lacks important enzymes for ketolysis, but are instead released into the circulation for metabolism in extrahepatic tissues. Rodents on a calorie-restricted diet increase gene expression of HMGCS2 in the liver, and in cases where the calorie-restricted diet is ketogenic, upregulations of HMGCS2 are also found in brain tissue [22].

### 2.2. Ways of Increasing the Concentration of Circulating Ketone Bodies

Besides fasting, ketogenic diets and nutritional ketogenic supplements can increase the circulatory pool of ketone bodies. The ketogenic diet mimics the metabolic profile of fasting, due to the high consumption of lipids and very low intake of carbohydrates, which is usually restricted to less than 30–50 g/day [23]. This relatively low energy contribution from carbohydrates enhances beta-oxidation and induces ketosis, in part owing to the low stimulus on beta-cells to induce insulin secretion. A quite high level of ketosis (4.8 mM) has been achieved previously by individuals on a classic ketogenic diet (lipid to protein + carbohydrate ratio of 4.5:1) [8]. However, adherence to ketogenic diets is difficult to accomplish and often poor [24]. Furthermore, ketogenic diets may be associated with adverse effects such as gastrointestinal problems, weight loss (which often should be avoided in neurodegenerative diseases), hyperlipidaemia, and may also result in vitamin and mineral deficiency, due to the strict diet regime [25]. Thus, ketogenic supplements may be more applicable in some patient groups.

Dietary supplements of MCFA also induce ketosis. This happens relatively soon after ingestion, as this group of fatty acids is absorbed directly into the portal vein instead of the lymphatic system (in contrast to long-chain fatty acids) and is consequently rapidly converted to ketones via beta-oxidation in the liver [26]. The most ketogenic MCFA are caprylic acid (C8) and capric acid (C10), which are found in coconut oil and palm kernel oil [27]. However, the concentration of these lipids is relatively low in the oils and interventions with coconut oils only raise ketone body levels modestly [28]. Products with higher concentrations of caprylic and capric acid have been developed and induce a ketosis of ~0.6 mM [29].

Another alternative to the ketogenic diet is intake of exogenous ketone esters and salts, which significantly increase ketone levels to >1 mM post ingestion, with the ketone ester being most potent in raising circulating ketones even while consuming regular meals [30,31]. Ketone salts often consist of a mixture of the D- and L-isoform of BHB, though the metabolic contribution of the L-isoform is poorly understood. All three approaches (ketogenic diet, MCFA, and exogenous ketone bodies) have been used in studies of neurodegenerative diseases, with most employing MCFA. It should be noted that MCFA may possess neuroprotective effects not related to ketonemia, since MCFA are able to cross the blood–brain barrier (BBB) and function as substrates for energy metabolism [32]. Furthermore, studies indicate that the MCFA, capric acid, may have the ability to improve mitochondrial function and reduce neuronal hyperactivity, which is often seen in AD, by inhibiting glutamatergic AMPA receptors [32].

### 2.3. Ketone Bodies Entering the Brain via Monocarboxylate Transporters

The uptake of ketone bodies across the BBB is carrier-dependent and unlike glucose transport, not increased by neuronal activity, but instead related to concentrations in the circulation [10]. MCTs are the only known transporters for ketone bodies and are distributed throughout the brain [9]. Studies in rodents have revealed that MCT expression is quite distinct between cell types, with the MCT1-isoform located at the BBB on endothelial cells and astroglia [9]. Further, astrocytes also express MCT4, which like MCT1 has a relatively low affinity for BHB [33]. In contrast, neurons almost exclusively express the MCT2 isoform, which possesses a high affinity for BHB [33]. The expression of MCT2 in neurons is co-localized to the mitochondria-rich postsynaptic density structures [34], suggesting that the transporter may play an important role in synaptic transmission [9]. Together this suggests that neurons, and to a certain extent also astrocytes, have the capacity to take up ketone bodies. Studies on tissue samples report that the MCT1 and 2 expression is quite similar in human brains compared to animal models [35,36].

The transport capacity of ketone bodies over the BBB in rodents is up-regulated by fasting [37], which may also be the case in humans [38]. The enhanced capacity is likely due to an upregulation of MCT expression, which is found in rodents both after a ketogenic diet [39,40] and after exercise [41].

### 2.4. The Catabolism of Ketone Bodies in Neuronal and Glial Cells

Once BHB and acetoacetate have been transported into the brain, they are converted back into acetyl-CoA, which enters the TCA cycle for ATP generation (Figure 1; neuron). The conversion occurs inside the mitochondria where BHB is transformed into acetoacetate by the reversible action of BDH using NAD+ and subsequently forming NADH. Acetoacetate is catabolized to acetoacetyl-CoA by succinyl-CoA:3-ketoacid coenzyme A transferase (SCOT). mRNA levels of SCOT are detectable in all human tissues excepts the liver [42]; therefore, the liver is unable to utilize ketone bodies as an energy substrate. Acetoacetyl-CoA is then converted back into two acetyl-CoAs ready to enter the TCA by the reversible action of thiolase (the first enzyme of ketogenesis). In contrast to glucose, this conversion of BHB and acetoacetate into an oxidizable form does not require ATP [20,43]. In the developing rodent brain, cultured neurons, astrocytes and oligodendrocytes were all able to utilize ketone bodies for oxidative metabolism at rates far greater than they utilized glucose. However, neurons and oligodendrocytes seemed to be more efficient in oxidizing ketones than astrocytes [44].

### 2.5. Glial–Neural Compartment Mode for Ketone Body Supply to Neurones

Glycolysis in astrocytes results in lactate production, supplying a source of energy for neurons, termed the astrocyte neuron lactate shuttle [45]. Further, fatty acids may cross the BBB, thereby reaching astrocytes closely connected to neuronal structures [46]. In astrocytes, degradation of fatty acids results in ketone body release to neighbouring neurons [47]. Thus, not only exogenous, but also endogenous ketone bodies can serve as fuel for the brain (Figure 1; astrocyte). MCFA supplementation may cause different modulations to astrocyte metabolism, which either enhances astrocyte neuron lactate shuttle or accelerates ketogenesis in astrocytes depending on the MCFA applied [48]. Endogenous production of ketone bodies is regulated by AMP-activated protein kinase (AMPK) activity [47]. Like elsewhere in the body, AMPK may be stimulated by 5-aminoimidazole-4-carboxamide ribonucleotide (AICAR) [49] and metformin [50], leaving a potential for pharmacological interventions. Finally, AMPK-activated ketogenesis in astrocytes is stimulated by both hypoxia [49] and hypoglycaemia [47].

## 3. The Effect of Ketone Bodies on Brain Metabolism

### 3.1. Ketone Bodies Affect Brain Metabolism in Healthy Individuals

In humans, both acute and chronic increases in ketone body availability to the central nervous system cause massive changes in cerebral fuel metabolism. In healthy middle-aged subjects, an i.v. infusion of BHB caused approximately 14% decrease in cerebral glucose consumption while oxygen use was unchanged, suggesting that ketone bodies, even when supplied acutely, enter the brain and may be utilized immediately as an alternative fuel to glucose [51]. Similar cerebral metabolic changes have also been demonstrated in young individuals [52], however, at lower infusion rates, and thus lower BHB concentrations, glucose uptake does not seem to be affected [53]. Together, this suggests an acute cerebral glucose-sparing effect when ketone availability is high. Comparable metabolic adaptations, where ketones substitute glucose, have also been reported in individuals on ketogenic diets [8]. During prolonged fasting, a more pronounced shift from glucose to ketone body metabolism is reported with acetoacetate and BHB supplying more than 50% of the brain’s energy [54], thereby replacing glucose as the main fuel supply. Availability seems crucial to cerebral uptake of ketone bodies during acute infusions in humans [55], whereas animal studies suggest that adaptation to increased levels of ketone bodies during fasting is accompanied by an increase in the brain’s capacity for ketone uptake, at least partly explaining the vast increase in ketone uptake after prolonged fasting [37,56]. Thus, both acute and chronic exposure to ketone bodies will increase the availability of alternative fuels for the brain.

During resting conditions, ketone bodies replace other energy sources rather than supplement them, resulting in unchanged ATP levels in the brain of healthy individuals [57]. However, other metabolic adaptations may occur both acutely and after longer interventions. Bough et al. found that rodents fed a calorie-restricted ketogenic diet, compared with an ad libitum control diet, for 3 weeks exhibited an upregulation of transcripts encoding proteins related to energy metabolism, including mitochondrial proteins for oxidative phosphorylation. In addition, the ketogenic diet enhanced mitochondrial biogenesis, and elevated the phosphocreatine/creatine ratio in the hippocampus [58], subsequently improving hippocampal metabolism. Like in the acute setting in humans, the metabolic adaptations did not result in detectible changes in ATP levels. When glucose availability is acutely reduced by experimental hypoglycemia, additional provision of ketones either by infusion or ingestion of MCFA preserves cognitive functions in patients with type 1 diabetes and healthy individuals, and increases the glycaemic threshold for symptoms and the counter-regulatory hormone response (i.e., greater hypoglycemia was required for initiation) [59,60]. This suggests that ketone bodies are not only able to save glucose, but also support brain metabolism during energy crises without prior adaptations from fasting. Hence, treatments that elevate circulating ketones are suggested to have implications in disorders characterized by compromised glucose metabolism, such as neurodegenerative diseases.

### 3.2. Hypometabolism in Neurodegenerative Disease is Ameliorated by Ketone Bodies

Most neurodegenerative diseases, including AD, PD, HD and ALS, are characterized by metabolic disturbances, which may be involved in both the development and progression of the disease [18]. The neurodegenerative diseases share several pathologies, such as protein aggregation, mitochondrial dysfunction, oxidative stress, neuroinflammation and more (see [61] for review), which may be induced by—or trigger—energy crisis. Another common feature in neurodegenerative diseases is the loss of specific neurons (AD: pyramidal neurons in Ammon’s horn of the hippocampus; PD: dopaminergic neurons from substantia nigra pars compacta in the basal ganglia; HD: enkephalin-positive medium spiny neurons from striatum in basal ganglia; ALS: fast-fatigable motor neurons in the spinal cord). These selective neurons have a high energy demand due to their long-range neuronal projections and extensive synaptic connections, making them more susceptible to metabolic disturbances [61]. In both AD, PD, HD, and ALS, glucose uptake and/or consumption is compromised [14,15,16,17,62]. Hypometabolism may be caused by a reduced density or activity of terminal neuronal fields and/or reduced glial metabolism [63] or by a reduced transport capacity [64]. Especially in AD, the glucose consumption monitored by ^18^F-FDG PET uptake correlates closely with cognitive performance and may be used as part of the diagnostic procedure [65]. In addition, fuel shortage is prominent even years before any cognitive impairment may be diagnosed [66], a phenomenon possibly mirroring the insulin resistance seen in obesity and type 2 diabetes where similar reductions in cerebral glucose consumption are seen [67,68,69].

The physiological response to energy shortage, as seen during fasting, is fat degradation and ketone body formation, thereby supplying fuel to the brain [7] and saving protein (the main source of gluconeogenesis) [63]. Supplying exogenous ketone bodies thus represents an intriguing means to mitigate cerebral metabolic disturbances which are found in most neurodegenerative diseases. Notably, and contrasting to glucose, the cerebral metabolism of ketone bodies remains unchanged in early AD when comparing patients to healthy individuals [70,71]. Additionally, exogenous supplies of ketone body donors that cross the BBB, particularly MCFA, may facilitate endogenous, intracerebral ketone body formation mediated by astrocytes [72]. Recently, it was reported that elevated ketone levels improve total energy metabolism in humans. Fortier et al. [73] demonstrated that MCFA supplementation for 6 months could enhance cerebral ketone metabolism without affecting the metabolic rate of glucose, thus increasing total energy metabolism in patients with mild cognitive impairments. Further, this intervention tended to improve some cognitive measures. Similar metabolic improvements have been demonstrated in patients with AD after 1 month of MCFA (both C8 and C10) supplementation [74].

### 3.3. Ketone Bodies May Support Metabolism Besides Being a Substrate

Disturbed glucose metabolism in association with oxidative stress is present early in disease progression of neurodegenerative diseases. This may trigger other abnormalities, such as reduced anti-oxidant defence and aggregation of misfolded proteins, thereby promoting further metabolic crisis and oxidative stress, eventually creating a vicious circle [16]. Furthermore, neuroinflammation is a common feature in neurodegenerative disease and may promote energy crisis [75]. Oxidative stress is characterized by an accumulation of reactive oxygen species (ROS), due to overproduction or diminished elimination [20]. Ketone bodies may regulate ROS balance through direct and indirect pathways. This is supported by in vitro studies, showing that ketone bodies may function as direct antioxidants, suppressing mitochondrial ROS production and promoting transcriptional activity of the antioxidant defence [76]. Haces et al. [77] demonstrated that both the L- and D-isoform of BHB and acetoacetate possess direct scavenging effects on ROS (BHB effects were specific to hydroxyl radicals). The mechanisms by which ketone bodies possess direct antioxidative effects remain to be elucidated, but the higher capacity related to BHB was speculated to be related to its hydroxyl group [77]. Another in vitro study in neocortical neurons and isolated mitochondria, showed that a combination of BHB and acetoacetate reduced ROS production stimulated by calcium induced glutamate excitotoxicity, and that this effect was due to enhanced NADH oxidation and thus, an increased NAD+/NADH ratio [78]. Both the increased NAD+/NADH ratio and the BHB itself may stimulate the antioxidant defence system through activation of different transcription factors [76,79].

In rodents fed an ad libitum ketogenic diet, hippocampal NAD+/NADH levels increased significantly within the first two days of the diet and remained elevated after 3 weeks of following the diet [80]. Likewise, an acute increase in the cerebral NAD+/NADH ratio has been reported in young healthy subjects shortly (45 min) after ingesting an MCFA product [57]. However, it is not known if this observation relates to any changes in antioxidant capacity or clinical effect in humans. Not all studies support that ketone bodies improve antioxidant capacity [81]. Oxidative stress may lead to mitochondrial dysfunction, further increasing production of ROS [79]. Mitochondrial efficiency can be enhanced by ketone bodies, probably through the expression of uncoupling proteins (UCP) [79]. Experimental studies in rodents have demonstrated that animals fed both a ketogenic diet or a ketone ester upregulate UCP4 and 5 in the brain [82,83]. Neuronal UCPs are important for the regulation of ROS production by reducing the mitochondrial membrane potential, thus normally leading to lower ATP generation [84]. However, it appears that UCPs activate mitochondrial proliferation in neurons and thereby compensate for the lower ATP production per mitochondria [84]. Furthermore, several studies indicate that BHB possesses anti-inflammatory effects [79], where animal models of PD [85] and AD [86] have been used to report reductions in microglial activation with ketone administration. Hence, these preclinical studies suggest that ketone bodies may support energy metabolism in ways not limited to fuel supply.

**Figure 2:**
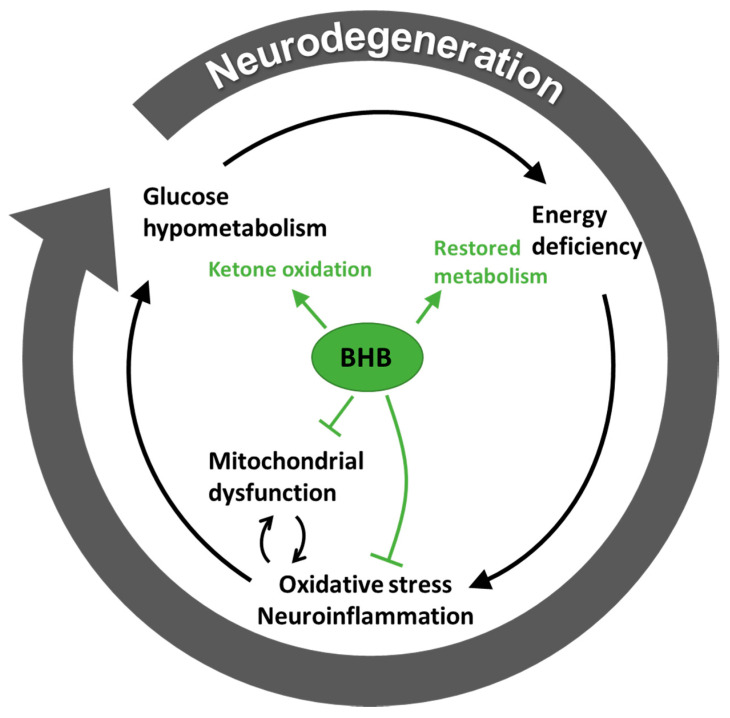
Vicious circle of energy crisis in neurodegenerative disease. The proposed effects of beta-hydroxybutyrate (BHB) on disease mechanisms are illustrated in green, demonstrating an inhibition of oxidative stress, neuroinflammation and mitochondrial dysfunction together with a facilitated ketone oxidation, which results in at least a partially restored metabolism.

The theory related to the fuel crisis seen with neurodegenerative disease is summarised in Figure 2, which also demonstrates the proposed protective mechanism of ketone bodies, especially BHB.

## 4. The Therapeutic Role of Ketone Bodies in Neurodegeneration

### 4.1. Ketone Bodies and Cognition in Alzheimer’s Disease and Related Conditions

A number of approaches to increase ketone body availability have been applied in elderly individuals and in diseases affecting cognition. These approaches include both acute interventions of BHB infusion [87] or a single meal/drink of MCFA [88,89,90], and also long-term treatments with continued MCFA supplementation as an add-on to the regular diet [91,92,93,94,95,96] or complete conversion to a ketogenic diet [97,98,99]. A predominance of studies has been conducted in patients with mild cognitive impairment and AD and apply the MCFA supplementation approach to achieve ketonemia. Hence, future studies implementing other ketogenic approaches will strengthen the evidence of a ketone-induced cognitive improvement.

Individuals without cognitive disease: In persons without known cognitive disease but suffering from type 2 diabetes, which is a risk factor for cognitive impairment [100,101], an i.v. infusion of BHB (blood levels of 2.4 mM), significantly improved working memory performance when compared to a placebo infusion. However, no change in a global cognitive score was found [87]. Further, in elderly persons without dementia, a single ketogenic meal of MCFA improved performance in several cognitive domains, with improvements correlating to levels of circulating BHB [89]. Consistently, cognitive benefits have been reported in elderly nursing home residents with prolonged treatment durations of MCFA supplementation [95]. These studies together suggest that individuals without cognitive disease may benefit from a ketogenic intervention. Notably, increased age and type 2 diabetes (both characterized by insulin resistance) significantly increase risk of AD, and both have been associated with cerebral glucose hypometabolism [10]. Similar to AD, ketone metabolism does not decline with aging [102].

Individuals with cognitive impairment: In patients with mild cognitive impairment and AD, a single acute dose of MCFA resulted in significant increases in BHB (from 0.04 mM to 0.52 mM), Reger et al. [88] found improved ADAS-Cog score and paragraph recall in a placebo-controlled cross-over design. Subsequent analysis found this to be true for ApoE4-negative subjects only [88]. In contrast, a quite similar experiment including twenty Japanese patients with mild-to-moderate AD, did not find any acute improvement in any cognitive measure after ingestion of MCFA [90]. No genotyping was performed in this study, which could explain the absent effect. Since an acute cognitive improvement was found in three studies, it could imply that increased energy supply during ketone treatment, partly explains the neuroprotective effect, since other signalling properties of ketone bodies might be expected to require more time.

In a non-acute (exposure 90 days) placebo-controlled trial of an oral ketone supplement (AC-1202; composed of caprylic acid) in 152 patients with mild to moderate AD, significant improvements in cognitive performance, evaluated by the ADAS-Cog score, were reported [91]. In this trial, BHB levels increased (two hours after dosing) to an average of ~0.4 mM after three months intervention, and similar to previous studies, the mitigating effects of the ketone body supplement was limited to ApoE4 negative participants [91]. Likewise, a cross-over study consisting of thirty days MCFA supplementation in AD patients, also resulted in an improvement in the ADAS-Cog-C score in ApoE4 negative patients and not in ApoE4 positive (only three individuals were ApoE4 positive) [92]. The reason for this genotype-specific effect of ketogenic treatment is yet unknown but has been speculated to be derived from differences in the pathophysiology between ApoE4 positive and negative [88,91]. For instance, mitochondrial function differs between genotypes, being lower in ApoE4 positive individuals, and thus may limit the utilization of ketones. Another proposed mechanism, is that the slightly more pronounced insulin resistance in ApoE4 negative individuals, upregulates the expression of MCTs, which is elevated in diabetes, allowing for a higher uptake and thus, utilization of ketones [91].

In agreement with the above studies, Fortier et al. [93] recently published the cognitive outcomes from the BENEFIC trial, a 6 month placebo-controlled trial of MCFA supplementation in patients with mild cognitive impairment (MCFA arm: *n* = 39; placebo arm: *n* = 43). The study found significant improvements in tests related to memory (free and cued recall), executive function (verbal fluency and Trail-Making Test), and language (Boston Naming Test), which may be related to the improved total energy metabolism in the brain found in the first part of the trial [73].

The largest trial conducted so far applying a ketogenic intervention was recently published by Henderson et al. [94]. In this study, 413 patients with mild-to-moderate AD were randomized to receive either a new ketogenic formulation (AC-1204) or placebo for 26 weeks to assess the safety and efficacy of this compound. Results were disappointing with regard to the cognitive evaluations; no effect was found on the ADAS-Cog score compared to placebo for either ApoE4-positive or negative patients. Only modest elevations of ketone levels were seen, which corresponded to less than half of the increase found with previously used compound (AC-1202). This level of ketonemia is most likely insufficient to improve cerebral metabolism and further suggests that some increase in ketone body levels is necessary to achieve a cognitive improvement [94].

**Figure 3:**
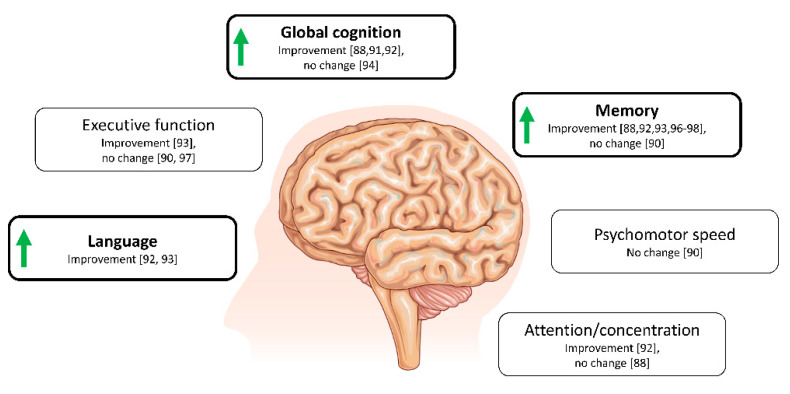
Overview of cognitive domains affected by ketogenic interventions in patients with mild cognitive impairment or AD. Overall improvements are demonstrated by green arrows. Illustration is solely based on studies using a randomized-controlled study design (cross-over or parallel groups). Interventions included ketogenic diets [97,98] or supplementation with MCFAs [88,90,91,92,93,94,96] ranging from acute (90 min after ingestion) to 6 months in duration and studies include between 12 and 413 participants.

Diet-induced hyperketonaemia (very low versus high carbohydrate diets) over six weeks was applied by Krikorian et al. [97] in a small sample of patients with mild cognitive impairment resulting in improved verbal memory performance in the low carbohydrate group. In the post-hoc analyses, the improvements were correlated to ketone body levels. No correlation was seen with other metabolic changes such as weight, insulin or glucose levels [97]. Similarly, in a series of studies with a limited number of participants, ketogenic dietary interventions significantly improved cognition in AD or mild cognitive impairment [96,98,99]. The potential cognitive benefit from exogenous ketones has not been described in detail. In a single case-study of a subject with early-onset AD ketone ester treatment was initiated, this resulted in improved memory retrieval, mood and performance of daily activities [103]. Overall effects on cognition are shown in Figure 3.

Apart from cognition, a number of paraclinical features have been studied in neurodegeneration. For example, the notion of disturbed cerebral fuel metabolism in neurodegeneration is far from recent; in 1960, Lassen et al. described a decreased cerebral oxygen metabolism in people with “presenile dementia”, a finding corroborated by Lying-Tunell et al. in 1981 [104,105].

In people with milder cognitive complaints, both Neth et al. [106] and Nagpal et al. [107] describe an increased amyloid content and a trend towards a lower TAU and a lower neurofilament light level after six weeks on a ketogenic diet. In the first of these studies, PET imaging showed an increase in acetoacetate metabolism with no change in glucose metabolism, whereas in the latter, the changes in cerebral spinal fluid markers could be related to ketone-induced changes in the gut microbiome. Similar results were described by Fortier et al. [73] in people with mild cognitive impairment. As for cerebral blood flow, in a study employing 45 days of exposure to caprylidene, a precursor for BHB and acetoacetate, patients with mild AD significantly increased blood flow in several brain regions, with the effects confined to people with no ApoE4 allele [108].

### 4.2. Ketone Bodies in Other Neurodegenerative Diseases

In PD, several animal studies have demonstrated neuroprotective effects of ketosis [109,110,111]. Only a few human studies have been conducted. In a pilot study, five patients were able to prepare a ketogenic diet and adhere to it for 4 weeks, which lead to significant improvement in the Unified Parkinson’s Disease Rating Scale scores [112]. Further, when compared to a regular diet, a ketogenic diet for 3 months improved the voice handicap index in PD patients [113]. Phillips et al. [114] randomized patients with PD to 8 weeks of a ketogenic diet versus a low-fat, high-carbohydrate diet. Both diet groups significantly improved in motor and non-motor symptoms; however, the ketogenic group showed greater improvements in non-motor symptoms, including cognitive function.

In animal models of ALS, exposure to hyperketonaemia alters clinical and biological manifestations of the disease. A ketogenic diet led to higher motor neuron survival and an improvement in motor function in a SOD1-G93A transgenic ALS model compared to controls [115]. The same research group has further demonstrated that supplementation of a precursor of ketone bodies alleviates ALS-motor impairment together with an increase in mitochondrial oxygen consumption rates in the spinal cord. This suggest that the additional ketones might be used as an alternate substrate for energy metabolism in ALS animals [116]. In vivo, BHB exerts dose-dependent neuroprotection, preserving motor function and reducing motor neuron loss [117]. This motor neuron protection is in agreement with results obtained in animal models with chronic AMPA-induced neurodegeneration, showing that BHB infusion prevents motor deficits and paralysis [118]. For HD, no conclusive effect of ketone bodies in animal models has been described [119,120,121].

## 5. Conclusions and Perspectives

Introducing ketone bodies for the treatment of neurodegenerative diseases may improve neuronal metabolism, which is hampered in such conditions. The observation that some individuals acutely (within 2 h) show improved cognitive function, suggests that ketones immediately provide additional or more efficient energy production in individuals with or at risk of neurodegenerative disease. With long-term ketogenic treatment additional adaptations might take place. Preclinical studies suggest that ketone metabolism may be enhanced by persistent ketonemia through increased MCT expression and that other adaptations influencing cerebral metabolism occur. However, these effects are most likely not disease modifying, since cognitive improvements disappear when ketogenic treatment is discontinued [91]. Small or medium-sized (*n* ≤ 150) clinical studies, mainly in AD, suggest a positive effect on a few disease outcomes, with most evidence demonstrating improvements in cognitive functions related to memory and language with ketogenic treatments in patients, who are already cognitively impaired. No definitive large-scale clinical studies are currently available. Several ways of introducing ketonemia in patients now exist and seem to yield comparative results. However, the most commonly used approach is MCFA supplementation, which—compared to the ketogenic diet and exogenous ketones—induces considerably lower levels of ketonemia. Interestingly, some studies have found a correlation between blood levels of ketone bodies and cognitive improvements, implying that treatments which significantly elevate ketone body levels could be more beneficial, but this hypothesis remains to be explored further.

Apart from ketogenic supplements and ketogenic diets, where implementing their use may be hampered by both availability and adherence problems, new drugs currently used for lowering glucose levels in type 2 diabetes—sodium glucose cotransporter 2 inhibitors (SGLT2-i)—increase circulating levels of ketone bodies to levels comparable to the ones achieved with MCFA supplements [122]. Indeed, in a pharmaco-epidemiological study, Wium-Andersen et al. [123] recently described a decreased risk of getting a dementia diagnosis while treated with an SGLT2-i compared to treatment with most other anti-diabetic drugs. Applying this drug class to induce mild ketosis could be a possible approach in further studies of neurodegenerative disease.

## Abbreviations

Acetyl-CoA	acetyl coenzyme A
AD	Alzheimer’s disease
AICAR	5-Aminoimidazole-4-carboxamide ribonucleotide
ALS	Amyotrophic lateral sclerosis
AMPK	AMP-activated protein kinase
BBB	Blood–brain barrier
BHB	beta-hydroxybutyrate
BHD	beta-hydroxybutyrate dehydrogenase
GLUT-1	glucose transporter type 1
HD	Huntington disease
HMG-CoA	3-hydroxy-3-methylglutaryl-CoA
HMGCS2	3-Hydroxy-3-Methylglutaryl-CoA Synthase 2
MCFA	medium-chain fatty acids
MCT	monocarboxylate transporter
PD	Parkinson’s disease
ROS	reactive oxygen species
SCOT	succinyl-CoA:3-ketoacid Coenzyme A transferase
SGLT2-i	sodium glucose cotransporter 2 inhibitors
TCA	tricarboxylic acid
UCP	uncoupling proteins
